# Up-Regulation of mRNA Ventricular *PRNP* Prion Protein Gene Expression in Air Pollution Highly Exposed Young Urbanites: Endoplasmic Reticulum Stress, Glucose Regulated Protein 78, and Nanosized Particles

**DOI:** 10.3390/ijms141223471

**Published:** 2013-11-28

**Authors:** Rodolfo Villarreal-Calderon, Maricela Franco-Lira, Angélica González-Maciel, Rafael Reynoso-Robles, Lou Harritt, Beatriz Pérez-Guillé, Lara Ferreira-Azevedo, Dan Drecktrah, Hongtu Zhu, Qiang Sun, Ricardo Torres-Jardón, Mariana Aragón-Flores, Ana Calderón-Garcidueñas, Philippe Diaz, Lilian Calderón-Garcidueñas

**Affiliations:** 1 Davidson Honors College, University of Montana, Missoula, MT 59812, USA; E-Mail: rodolfovillarreal36@hotmail.com; 2 Hospital Central Militar, Secretaria de la Defensa Nacional, Mexico City 11649, Mexico; E-Mails: mfrancolira@yahoo.com.mx (M.F.-L.); maragonf@hotmail.com (M.A.-F.); 3 Instituto Nacional de Pediatria, Mexico City 04320, Mexico; E-Mails: agonzalezmaciel@yahoo.com (A.G.-M.); reynosoraf@yahoo.com (R.R.-R.); betyepg@gmail.com (B.P.-G.); 4 The Center for Structural and Functional Neurosciences, the University of Montana, Missoula, MT 59812, USA; E-Mail: lou.herritt@mso.umt.edu; 5 Visiting Student, Ministry of Education of Brazil, Rio de Janeiro 20000-000, Brazil; E-Mail: larazevedo_2002@hotmail.com; 6 Division of Biological Sciences, the University of Montana, Missoula, MT 59812, USA; E-Mail: dan.drecktrah@mso.umt.edu; 7 Department of Biostatistics, Gillings School of Global Public Health, University of North Carolina, Chapel Hill, NC 27599, USA; E-Mails: htzhu@email.unc.edu (H.Z.); qsun@live.unc.edu (Q.S.); 8 Centro de Ciencias de la Atmosfera, Universidad Nacional Autonoma de Mexico, Mexico City 04510, Mexico; E-Mail: rtorres@unam.mx; 9 Instituto de Medicina Forense, Universidad Veracruzana, Boca del Rio 94297, Mexico; E-Mail: acald911@hotmail.com; 10 Core Laboratory for Neuromolecular Production, the University of Montana, Missoula, MT 59812, USA; E-Mail: philippe.diaz@mso.umt.edu

**Keywords:** air pollution, BiP, children, oxidative stress, endoplasmic reticulum stress, GRP78, sarcoplasmic reticulum, myocardial damage, nanoparticles, particulate matter, *PRNP*

## Abstract

Mexico City Metropolitan Area children and young adults exposed to high concentrations of air pollutants including fine and ultrafine particulate matter (PM) *vs.* clean air controls, exhibit myocardial inflammation and inflammasome activation with a differential right and left ventricular expression of key inflammatory genes and inflammasomes. We investigated the mRNA expression levels of the prion protein gene *PRNP*, which plays an important role in the protection against oxidative stress and metal toxicity, and the glucose regulated protein 78, a key protein in endoplasmic reticulum (ER) stress signaling, in ventricular autopsy samples from 30 children and young adults age 19.97 ± 6.8 years with a lifetime of low (*n*:4) *vs.* high (*n*:26) air pollution exposures. Light microscopy and transmission electron microscopy studies were carried out in human ventricles, and electron microscopy studies were also done in 5 young, highly exposed Mexico City dogs. There was significant left ventricular *PRNP* and bi-ventricular GRP78 mRNA up-regulation in Mexico City young urbanites *vs.* controls. *PRNP* up-regulation in the left ventricle was significantly different from the right, *p* < 0.0001, and there was a strong left ventricular *PRNP* and GRP78 correlation (*p* = 0.0005). Marked abnormalities in capillary endothelial cells, numerous nanosized particles in myocardial ER and in abnormal mitochondria characterized the highly exposed ventricles. Early and sustained cardiac ER stress could result in detrimental irreversible consequences in urban children, and while highly complex systems maintain myocardial homeostasis, failure to compensate for chronic myocardial inflammation, oxidative and ER stress, and particles damaging myocardial organelles may prime the development of pathophysiological cardiovascular states in young urbanites. Nanosized PM could play a key cardiac myocyte toxicity role.

## 1. Introduction

Air pollution is a significant health problem in megacities around the world. Short and long term exposures to particulate matter (PM) air pollution increase the risk for adverse clinical cardiovascular (CV) events as well as CV mortality [1–5]. Pulmonary and systemic oxidative stress and inflammation are critical intermediary pathways playing a role in the detrimental effects on the CV system in response to PM inhalation [1].

Metropolitan Mexico City residents are exposed year-round to air pollutant concentrations above the National Air Ambient Quality Standards (NAAQS) for the United States [6–8]. Concentrations above the standards for fine particulate matter (PM_2.5_) as well as significant levels of PM associated with lipopolysaccharides (PM-LPS) are present in Mexico City’s air, and marked regional differences in the air pollutants concentrations and composition have been reported within the Mexico City Metropolitan Area (MCMA) [9,10]. Well-established regional differences in air pollution between South and North MCMA have been assessed by our laboratory for differential health effects in mice exposed for 16 months to ambient air [11]. *Interleukin-1*β (*IL-1*β), *tumor necrosis factor-*α (*TNF-*α), and *cluster designation antigen 14* (*CD14*) gene mRNA myocardial expression were up-regulated in mice exposed to South *vs.* North MCMA [11]; significant differences were attributed to the regional differences in ambient air PM-LPS concentration [9,12]. We recently investigated the expression of myocardial inflammatory genes in right and left ventricles of 21 children and young adults ages 18.5 ± 2.6 years, from South and North MCMA [13]. A significant S *vs.* N right ventricle up-regulation of *IL-1*β (*p* = 0.008), *TNF-*α (*p* = 0.001), *IL-10* (*p* = 0.001), and *CD14* (*p* = 0.002), and a left ventricle difference in *TNF-*α (*p* = 0.007), and *IL-10* (*p* = 0.02) were observed. South MCMA right ventricles had significant up-regulation of *NLRC1*, *NLRC3* and of 29/84 inflammasome genes, including NOD factors and caspases [13]. We concluded residency within MCMA likely influences the differential expression of key inflammatory myocardial genes and inflammasomes in young urbanites.

The normal cellular isoform of the prion protein (PrP^C^) is encoded by the *PRNP* gene, the product is a conserved glycosylphosphatidylinositol-anchored cell surface protein expressed by neurons and other cells [14]. PrP^C^ is widely distributed in the central nervous system (CNS) and in diverse extra CNS tissues including the myocardium [15]. PrP^C^ has antioxidant properties [16,17] and in solution acts as a radical scavenger, an essential property for protection of astrocytes against oxidative stress [18]. In the context of chronic prion brain infections, prion propagation exacerbates an apoptotic pathway: mitochondrial dysfunction follows mis-localisation of SOD2 to cytosolic caspases, allowing its degradation. The end result is the marked decrease in the cellular capacity to maintain oxidative homeostasis resulting in cell death [19]. PrP also plays a key role in copper metabolism [20] and has functional importance in the protection against oxidative stress and metal toxicity [21].

We are particularly interested in the role that myocardial PrP^C^ may play against oxidative stress and hence cardioprotection [22–25]. Growing evidence indicates that PrP^C^ modulates ion channels, intracellular signaling pathways, and has a role in the contractile function of skeletal and smooth muscles [26–30]. The impact of particulate matter on endoplasmic reticulum stress (ERS), the ERS in the heart, and the relationship between the PrP^C^ up-regulation and the ER stress marker Glucose regulated protein 78 (GRP78), also called immunoglobulin heavy chain binding protein (BiP) [31–37], are at the core of our interest.

The first aim of this study was to evaluate the left and right ventricular differences in mRNA expression of PrP^C^ and GRP78/BiP between control subjects with a lifetime exposure to low concentrations of air pollutants *vs.* residents in a highly polluted megacity. The second aim was to establish if there are differences in the levels of expression of the selected genes between the left and right ventricles. Our third aim was based on the current ER literature [31–38]. ER stress is a key player in the up-regulation of PrP^C^, thus, in order to define ER stress in our samples, we combined the mRNA expression of our selected reticulum stress marker, the molecular chaperone GRP78/BiP with optimal electron microscopic investigation of ER in young dogs exposed to the same high concentrations of particulate matter as the MCMA children and young adults included in this study.

Our results identify PrP^C^ and GRP78 ventricular up-regulation in highly exposed young urbanites and a significant differential ventricular response to their megacity exposures. The presence of nanosized particles in the endoplasmic reticulum and mitochondria suggests that nanosized PM is a key player in the myocardial damage and the ERS seen in young urbanites. The critical function of the heart as a pump is associated with the need to maintain efficient cardiac function under both physiological and pathological conditions [39]. Homeostatic conditions are associated with a balance between ER protein folding capacity and demand, while under intense ERS signaling processes the targeted cell will go toward apoptotic cell death and thus, the ERS responses become a maladaptive process [34]. Highly complex systems maintain myocardial homeostasis; failure to compensate for chronic myocardial inflammation, oxidative and ERS and particles damaging critical myocardial organelles may prime the development of pathophysiological cardiovascular states in children and young adults residing in polluted environments.

## 2. Results

### 2.1. Study City and Air Quality

Mexico City is an example of extreme urban growth and accompanying environmental pollution [6–10]. The metropolitan area of over 2000 km^2^ lies in an elevated basin 2240 m above sea level surrounded on three sides by mountain ridges. Mexico City’s nearly 20 million inhabitants, over 40,000 industries, and 4 million vehicles consume more than 40 million liters of petroleum fuels per day, producing an estimated annual emission of 2.6 t of particulate and gaseous air pollutants [40]. Mexico City’s metropolitan area motor vehicles produce abundant amounts of primary fine PM, elemental carbon, particle-bound polycyclic aromatic hydrocarbons, carbon monoxide, and a wide range of air toxins, including formaldehyde, acetaldehyde, benzene, toluene, and xylenes [41–43]. The high altitude and tropical climate facilitate ozone production all year and contribute to the formation of fine secondary particulate matter. Air quality is worse in the winter, when rain is less common and thermal inversions are more frequent. Selection of subjects from southern Mexico City *vs.* northern Mexico City was made based on the significant differences between outdoor environments in “northern-industrialized” zones in comparison with “southern-residential” zones, which illustrate the contribution from the industry in the north [7,10,42]. Southern Mexico City residents have been exposed to significant concentrations of ozone, secondary tracers (NO_3_^−^) and PM-LPS, while northern Mexico City residents have been exposed to higher concentrations of volatile organic compounds (VOCs), PM_2.5_, and its constituents: organic and elemental carbon including polycyclic aromatic hydrocarbons, secondary inorganic aerosols (SO_4_^2−^, NO_3_^−^, NH_4_^+^), and metals (Zn, Cu, Pb, Ti, Mn, Sn, V, Ba) [7,8,10,42]. Recent studies on the composition of PM_2.5_ with regards to sites and samples collected in 1997 show that composition has not changed during the last decade [7].

#### Air Quality Data

Mexico City residents are exposed year-round to PM_2.5_ and ozone concentrations above United States’ National Air Ambient Quality Standards (NAAQS). The PM_2.5_ annual air quality standard of 12 μg/m^3^ has been historically exceeded across the metropolitan area, including the selected target areas (Table 1). In the selected period between 1997 and 2012 significantly higher levels of fine PM were observed in the North *vs.* South due mostly to industry and heavy truck traffic (*p* < 0.0001). During the dry season extending from November to May, PM_2.5_ levels as high as ~90 μg/m^3^ are common in the northern location.

### 2.2. Human Heart Histopathology

**Figure 1.**
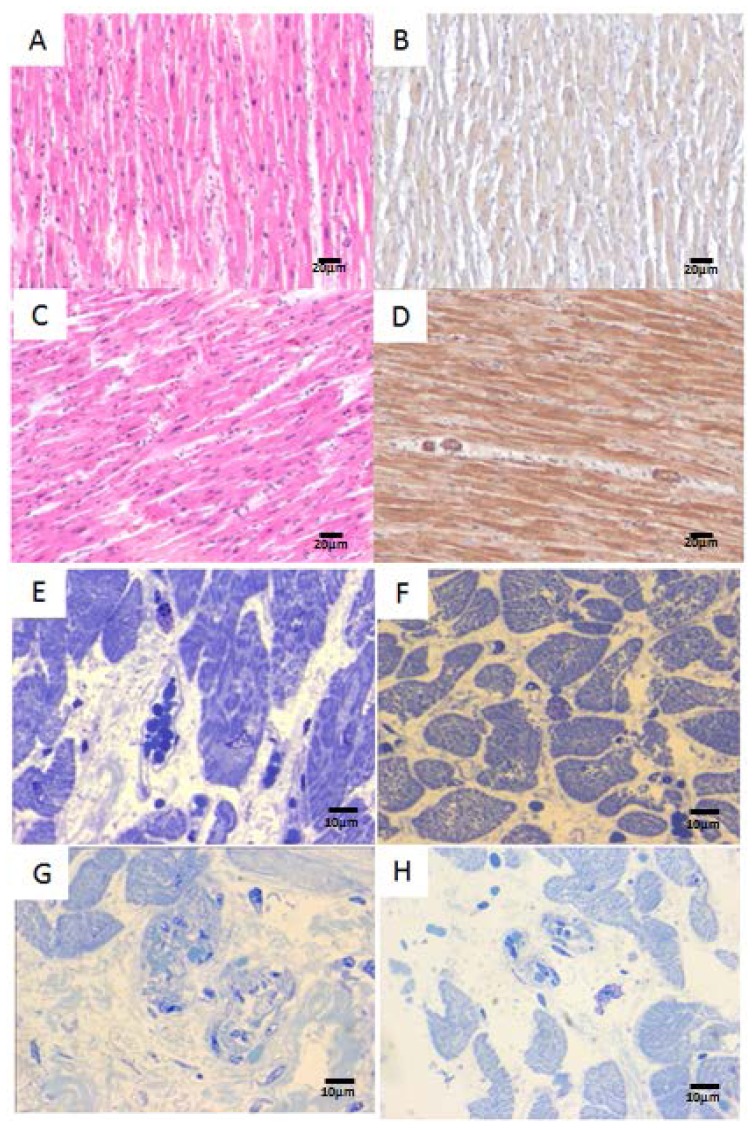
(**A**) The left ventricle in a 22 years old female control showing mild variation in nuclear size and unremarkable blood vessels. H&E ×40; (**B**) Same 22 years old control left ventricle with diffuse pale metallothionein immunostaining in myocardial fibers and negative staining in blood vessels, ×40; (**C**) Left ventricle in a 14 years old Mexico City girl with mild to moderate variation in nuclear size myocytes. H&E ×40; (**D**) Same exposed 14 years old girl left ventricle with metallothionein staining exhibiting stronger myocardial immunoreactivity, also involving endothelial and smooth muscle cells in arteriolar vessels, ×40; (**E**) A degranulated mast cell with clumping of nuclear chromatin is observed in this right ventricle from an 18 years old North MCMA male, Toluidine blue stain ×100; (**F**) In contrast, this 17 years control male shows intact fully granulated mast cells, Toluidine blue stain ×100; (**G**) Left ventricle in a 27 years old South MCMA male with sub-epicardial arterioles display thick walls, Toluidine blue stain ×100; and (**H**) Left ventricle in a 20 years male with unremarkable blood vessels, Toluidine blue stain ×40.

An average of 6 sections were stained and examined for each block, including hematoxylin-eosin and toluidine blue. Control hearts exhibited mild variation in nuclear size and blood vessels were unremarkable (Figure 1A,B). Mild to moderate variation in nuclear size in myocardial fibers (Figure 1C) were seen in Mexico City teens and young adults. Immunostaining for metallothionein show a diffuse pale staining in myocardial fibers of control subjects (Figure 1B), while exposed teens displayed stronger myocardial immunoreactivity, also involving endothelial and smooth muscle cells in arteriolar vessels (Figure 1D). Mast cell degranulation and mast cells with condensation of nuclear chromatin into sharply delineated masses that become marginated against the nuclear membranes were seen in the interstitial spaces in both right and left ventricles of highly exposed subjects (Figure 1E). Small sub-epicardial arterioles display thick walls in Mexico City young adults (Figure 1G). Intact mast cells, unremarkable blood vessels and no inflammatory myocardial or peri-vascular infiltrates were seen in low exposure subjects (Figure 1F,H).

### 2.3. Electron Microscopic Findings in Mexico City Dogs and Teens

**Figure 2.**
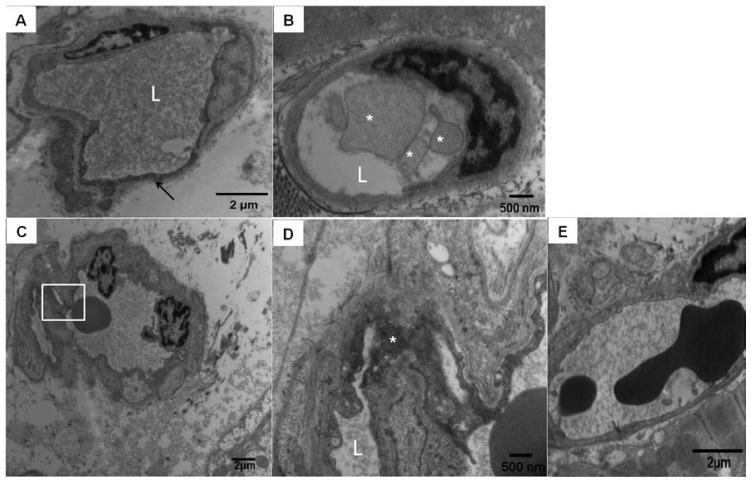
Mexico City dogs myocardial blood vessel pathology. (**A**) Scattered left and right ventricle capillaries exhibited endothelial cells with very dark cytoplasm, shrinkage, and partial detachment from the vessel lumen. This picture corresponds to a 4.6 years South MCMA animal facility male dog. “L” is marking the vessel lumen, magnification ×14,500; (**B**) South MCMA female dog with membranous luminal fragmented material in the vessel lumen (white *), magnification ×29,100; (**C**,**D**) correspond to a male 5 years old dog with numerous fine and ultrafine particulated material in the arteriolar wall (square), magnification ×7290; At ×29,100 (**D**), the conglomerates de PM are occupying the endothelial cytoplasmic space (white *); and (**E**) Normal left ventricle capillary in a 5 years old control dog, magnification ×14,500.

In contrast to human autopsy specimens, optimally fixed dog hearts permitted better assessment of ultrastructural myocardial changes. The electron microscopy performed in dog tissues showed isolated myocardial endothelial cells with very dark cytoplasm, shrinkage, and partial detachment from the vessel lumen (Figure 2A). These abnormal endothelial cells showed a significant number of cytoplasmic nanosized particles. Single particles measured 14 nm and conglomerates measured 28 and 55 nm. Some capillaries had membranous luminal fragmented material (Figure 2B). Small arterioles exhibited endothelial cells with numerous cytoplasmic intraluminal extensions and at higher magnification clusters of nanosized particles on average 28 nm were seen in association with increased pinocytic activity (Figure 2D). A normal capillary in a low pollution dog is seen in Figure 2E.

**Figure 3.**
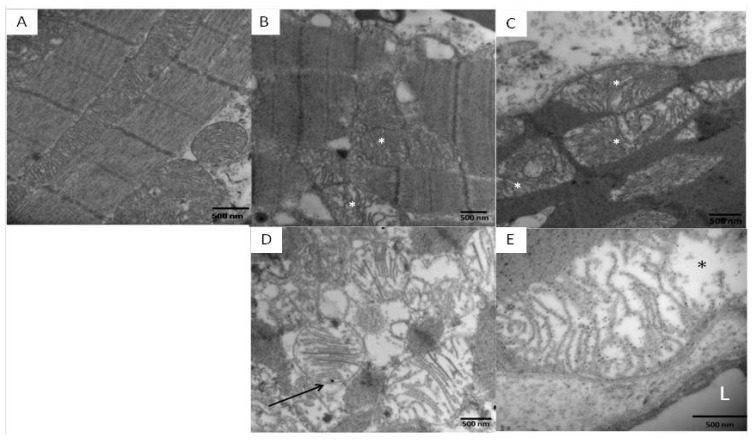
Mitochondrial images in left and right ventricles of control and South MCMA young dogs. (**A**) Unremarkable mitochondria and myocardial fibers in a 5 year old control left ventricle, magnification ×14,500; (**B**) Abnormal mitochondria had fragmented or missing cristae, magnification ×36,400; (**C**) Intra-mitochondrial lucent areas (white in **B**, **C**, and black in **E** *) are common in subsarcolemmal location, magnification ×43,700; (**D**) Abnormal mitochondria with intracristae nanosized PM (arrow), magnification ×43,700; and (E) Nanosized particles are seen in both abnormal cristae and in the midst of the electron lucent matrix, magnification ×72,900. Scale bar = 500 nm.

Marked abnormalities in mitochondrial morphology were seen in highly exposed dogs. Unlike control samples where the mitochondrial cristae are closely packed, uniform and linear (Figure 3A) [38], exposed dogs had fragmented or missing cristae (Figure 3B), with intra-mitochondrial lucent areas (Figure 3C) and an increase in fusion of multiple mitochondria producing giant mitochondria (Figure 3D). Numerous nanosized particles were observed attached to the abnormal cristae or in the midst of the electron lucent matrix (Figure 3D,E).

**Figure 4.**
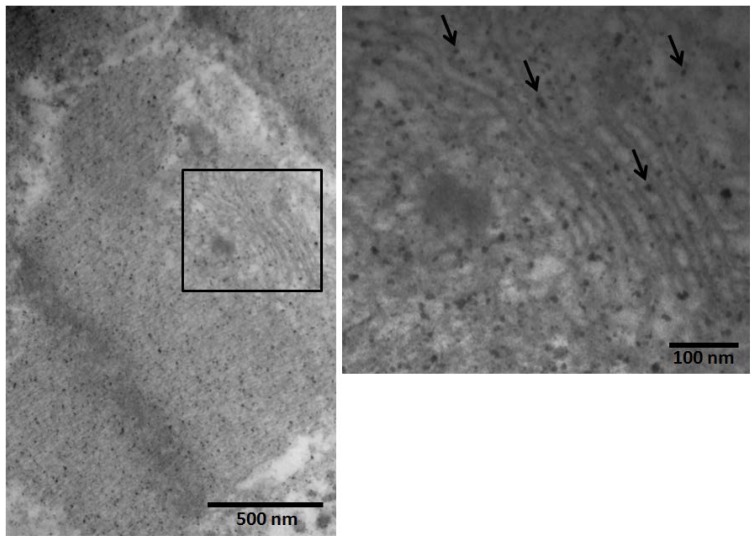
Extensive deposition of nanosized particles was seen in the left ventricular exposed myocardial endoplasmic reticulum of a Mexico City dog (in the inset, arrows point to the nanosized PM in the ER), EM magnifications ×50,000 and 120,000.

The endoplasmic reticulum (ER), a network of membranes responsible for secreted and membrane protein synthesis, protein processing, protein folding and lipid biosynthesis is a highly dynamic organelle with physical connection with mitochondria in cardiac myocytes [34,44]. The myocardial ER in Mexico City dogs showed the presence of nanosized particles in both left and right ventricles (Figure 4).

**Figure 5.**
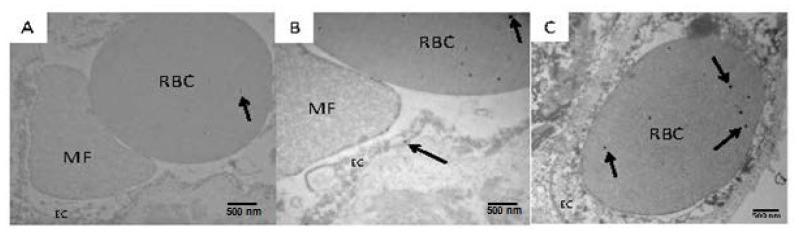
The integrity of myocardial cells was ill-preserved in human samples (**A**–**C**), however, nanosized particles could be seen in luminal red blood cells (RBC) (arrows on RBC in **A**–**C**) and in adjacent endothelial cells (EC) (arrow on EC in **B**) of an 11 years old MCMA girl (arrows point to the nanosized 14 nm in diameter particles). Luminal microfragments (MF) of unidentified cell origin are also common (**B**). Magnifications ×36,400. Scale bar = 500 nm.

Although the electron microscopic material obtained from human autopsies was not optimal, we were able to identify nano particles in luminal erythrocytes in myocardial capillaries (Figure 5).

### 2.4. Real-Time PCR Analysis of Target Genes

Real-time PCR analysis of PrP^C^ and GRP78/BiP in the heart samples indicated that the corresponding mRNA was present in each of the samples analyzed. The initial analysis included the relative quantization of gene expression controls *vs.* the Mexico City cases performed by the 2^−ΔΔ^*^C^*^(T)^ method including the quantification of the ΔΔ*C*_T_ and the fold change calculation (>2) according to the formula 2 Δ*C*_T_ (high pollution) – Δ*C*_T_ (low pollution). The difference in the cycle threshold (Δ*C*_T_) value was derived by subtracting the *C*_T_ value for GAPDH which served as the internal gene control, from the *C*_T_ value for BiP and PrP^C^. Once you have the Fold Change Calculation, the statistics are done taking into account fold changed values between controls and exposed subjects. Only gene expression values (≥2 fold controls *vs.* exposed) are considered significant. The mean fold increase in the expression of the selected markers PrP^C^ and BiP ranged from 0.72 to 6.8 compared to controls (Table 2). The *p* values of the PrP^C^ and BiP expression in left and right ventricles exposed *vs.* controls are seen in Table 2. PrP^C^ expression in the left ventricle of Mexico City residents is significantly different from controls (*p* = 0.006), while there is no difference on the right ventricle (*p* = 0.1360). For BiP, both right and left ventricles in exposed subjects are significantly different from controls. When we analyzed the left *vs.* right ventricle up-regulation PrP^C^ in Mexico City subjects, the left ventricle was significantly different from the right, *p* < 0.0001, while the expression of BiP did not show any significant differences between R and L ventricles (*p* = 0.1189).

The significance of the correlations between the PrP^C^ and BiP/GRP78 values in right and left ventricles is shown in Table 3.

We next separated residents in the North *vs.* South MCMA (Table 4). We used two-sample *t*-test and Wilcoxon rank sum test to explore the differences in the expression of PrP^C^ and BiP/GRP78 in the left and right ventricles based in Mexico City residency.

Finally, our analysis of the children cohort revealed no differences in gene expression between children regardless of area of residency (Table 5).

## 3. Discussion

Metropolitan Mexico City children and young adults exposed year round to high concentrations of air pollutants including fine particulate matter have significant mRNA upregulation of left ventricular PRNP prion protein gene and biventricular glucose regulated protein GRP78/BiP, when compared to low pollution controls. We found significant differences among exposed urbanites in the expression of PrP^C^ in left *vs.* right ventricles, the left ventricle showing a striking PrP^C^ up-regulation (*p* < 0.0001) and a strong positive correlation with GRP78/BiP (*p* = 0.0005).

The significant PrP^C^ left ventricular up-regulation is a key finding in highly exposed young urbanites given the multiple pathways impacted by the cellular form of PrP [22–25,31,38]. The antioxidant and metal interaction properties attributed to the PrP^C^ are particularly crucial in this scenario [16–23,25,26,31,38,45–51]. The critical relevance of the protective role of PrP^C^ has been discussed in the context of acute stroke and the pathogenesis of prion diseases [45–51]. Conversion of PrP^C^ to the pathogenic isoform PrP^Sc^ increases the vulnerability to oxidative insults and is a key downstream mediator of cellular stress-induced neuronal apoptosis [46,49,51]. Up-regulation of PrP^C^ protects against environmental neurotoxic metals-induced oxidative stress and apoptotic cell death [48]. In Déry *et al.*, breast cancer work, mRNA levels of GRP78/BiP correlated with PrP transcript levels in breast cancer tissues and breast carcinoma cell lines [31]. Endoplasmic reticulum stress was a positive regulator of *PRNP* gene transcription breast cancer cell lines [31]. This work is very relevant to ours, given the demonstration that in breast tissues PrP delayed ER stress-induced cell death [31]. Since the ER stress-mediated increase in PrP levels is associated with increased cellular survival in human breast cancers, the results are favorable to the cancer cells, but not to the patient [31]. The implications of high PrP^C^ in the ventricles of children and young adults with significant exposures to air pollutants could follow the route of the ER stress described upon exposure to nanoparticles and outlined in Christen and Fent work [38]: either the cell can cope with the stress and restore normal cellular functions, or it will undergo apoptosis. Apoptosis is not a welcome response in myocardial tissues, particularly because there is significant ventricular inflammation and inflammasome activation in highly exposed young MCMA residents [13]. It is plausible that the significant left *vs.* right ventricular PrP^C^ up-regulation is indeed a protective mechanism to a critical pump ventricular chamber with marked differences in morphologic and contractile properties and in myocardial microcirculation *vs.* the right chamber [52,53]. Pro-inflammatory mediators, including TNF-α and IL-1β have been implicated in the pathogenesis of myocardial dysfunction and cardiomyocyte death in ischemia-reperfusion injury, sepsis, chronic heart failure, viral myocarditis, and cardiac allograft rejection [54–57]. TNF-α is strongly up-regulated in the left ventricle of South MCMA residents, alongside IL-10 [13], thus, the possibility of a compensatory PrP^C^ left ventricular response in young ages when CV clinical effects are not yet present, would have to be entertained.

The endoplasmic reticulum is critical for proper cellular function and disruptions in ER are associated with a number of human diseases including CV pathology [32–37,39,58,59]. Given the critical myocardial function of ER as a major integration site of cell growth signaling and its association with the sarcoplasmic reticulum (SR), and their combined role in cardiac myocyte contraction, proper growth and metabolism, the need to maintain a healthy ER is imperative for proper myocardial function [32–35,39].

The issue of severe ER stress is very important in the context of PM exposures and nanosized particles [38,60–63]. Nanosized particles (<100 nm), pose a significant health risk through dermal, inhalation and oral routes [63]. Fine (<2.5 μm) and ultrafine (<100 nm) PM exert detrimental effects by induction of oxidative stress, DNA damage and apoptosis, inflammation, endothelial damage, arterial vasoconstriction, alterations in mitochondrial and myofibrillar structure among others [60–63]. Diesel exhaust ultrafine PM and titanium dioxide nano PM can directly induce cardiac cell damage through the formation of ROS and alterations of the myofibrillar structure [61]. The mitochondrial damage, ERS and cell damage with induction of apoptosis and necrosis are an intrinsic part of the ROS overproduction nano PM cytotoxicity [61–63]. Perturbations of calcium homeostasis are also the result of acute and chronic exposures to nanosized PM [64]. The nanosized PM alteration in calcium signaling mechanisms [64,65] is undoubtedly of key myocardial importance in the air pollution scenario, as is the fact that positively charged, amine modified polystyrene nanoparticles exhibit cytotoxic effects upon cardiomyocyte membranes producing calcium ion disturbances and cell death [66]. Of utmost relevance for highly exposed urbanites is the observation that these charged nano PM cause delayed depolarizations, reduction in conduction velocity and abnormal action potential duration in cultured neonatal rat cardiomyocytes [66]. These experimental observations relate closely with our Holter observations of supraventricular arrhythmias and decreased cardiac vagal tone in MCMA clinically healthy children [67]. Doroudgar and Glembotski [34] discussed an issue of paramount importance for megacity residents: calcium transfer from the ER to mitochondria (both abnormal and with nano PM in exposed MCMA residents) can regulate mitochondrial metabolic function *in vivo* [68–70]. The ERS can increase the extent of ER/mitochondria tethering [34,44], resulting in altered cellular and ER calcium dynamics that impact calcium cycling and could give rise to arrhythmias [67]. To complicate matters further in urban children, we are repeatedly observing ventricular degranulated mast cells, providing a key pathway for alteration of autonomic neurotransmission [71] and in modulation of cardiac contractility and the likelihood of arrhythmias through both endothelin-1 and myocardial mast cell degranulation [72].

Although our previous human and mice work [11,13] showed a strong difference of expression of inflammatory genes and inflammasomes in North *vs.* South MCMA residents, the results of the selected genes in this study showed a uniform up-regulation regardless of residency within MCMA. The fact that the left ventricle exhibited striking ER and mitochondrial abnormalities likely relates to the left morphologic and contractile properties [52] and differences in myocardial circulation [53]. These contractile and microcirculatory changes might contribute to the sharp differences between ventricular gene expression in subjects exposed to high levels of pollution.

Myocardial inflammation, up-regulation of several inflammasome components in order to assemble functional inflammasomes, and the central role of the NLR family as pathogen sensors and activators of inflammatory caspases and transcriptional regulation of immune response genes, including pro-inflammatory cytokines with detrimental cardiac effects, compounds the issue of the long-term impact of the innate immune altered responses, inflammation, ER stress and PrP^C^ up-regulation upon the cardiovascular system of highly exposed young individuals [11,13,73–75].

Oxidative stress and chronic inflammation lead to an increase in cardiovascular disease risk [1–3]. Our findings in seemingly healthy children and young adults provide important mechanistic pathways to explain the higher risk of cardiovascular disease in susceptible urban populations. A prime example is the outcome of cardiac ischemic events in urban residents, whose results depend strongly not only on the intensity and duration of the ischemic stimulus but also on the myocardial intrinsic tolerance to ischemic injury, even in the absence of manifest cardiovascular disease [76]. Thus, the concept of occult cardiotoxicity as described by Golomb *et al.* [76] should be taken into account in subjects with severe ER stress exposed to significant concentrations of air pollutants. A clear understanding of the adaptive and maladaptive ERS myocardial responses [34] is critical for cardioprotection of exposed individuals [11,13].

While recognizing that the study group is small, the endpoint results are nonetheless significantly different to warrant that residency likely plays a key factor in the myocardial PrP^C^ and GRP78/BiP responses. Additional characterization of the myocardial protein PrP^C^ and GRP78/BiP quantification and the full characterization of the particle-like material observed in myocardial fibers, blood vessels and red blood cells by energy filtered TEM [77] would have benefited these studies. This is important because combustion associated metals [10,38,49,51,65] are likely present in the ultrafine PM and could be responsible for the significant ER stress.

## 4. Experimental Section

### 4.1. Heart Samples

The IRB Committees of the involved institutions approved the study and the research protocol involving deceased individuals, while the Institutional Animal Care and Use Committee (IACUC) approved the canine study. Thirty clinically healthy, non-smoking, non-obese children and young adults who died suddenly, accidentally, and without chest or head trauma were included. Four subjects were residents of low polluted cities (1 F, 3 M), ages 14, 17, 20, 22, (18.25 ± 3.5 years), while 26 subjects (5 F, 21 M) were residents in MCMA (21.7 ± 10.17 years), including 11 residents in South Mexico City and 15 in NMC. Their major everyday activities, including work and school took place within 10 miles of their residency. Autopsies were performed 3.7 ± 1.7 h after death. Subjects had no pathological evidence of recent or long-term inflammatory processes or pathological findings such as myocardial infarction, valve pathology, coronary artery disease, ventricle or atrial dilatation or hypertrophy, large vessel gross abnormalities, chest trauma, cerebral ischemia, head injury, or stroke. Toxicological studies were negative and included drug alkaline and acid/neutral screen, amphetamines, benzodiazepines, cocaine/opiates, alcohol, volatiles and cannabinoids. All subjects were negative for the Asp299Gly TLR4 polymorphism. The mean age of the Southern Mexico City subjects was 23.91 ± 9.92 years (mean ± standard deviation (SD)) and 20.19 ± 10.38 years for the Northern Mexico City subjects (*p* = 0.38). Representative sections of the heart muscle including the left and right ventricles and the inter-ventricular septum were fixed in 10% neutral formaldehyde for 48 h and transferred to 70% alcohol for histopathology. Heart tissues were fixed in 2% paraformaldehyde and 2% glutaraldehyde in sodium phosphate buffer (0.1 M, pH 7.4) for electron microscopy. The remaining heart tissues were quickly frozen and stored at −80 °C and transmural sections of the left and right ventricular wall were selected for the RT-PCR studies.

Given the electron microscopy poor preservation of the human myocardial tissues, we selected to use myocardial optimally fixed electron microscopy tissues from 5 young dogs (<5 years) from an independent longitudinal study involving the use of Nimesulide^®^ in mixed beagle dogs. The 5 selected dogs for this study were non-treated Mexico City dogs exposed 24/7 to the South MCMA atmosphere. Procedures used were in accordance with the guidelines of the Instituto Nacional de Pediatria (INP) on the Use and Care of Laboratory Animals. The INP provided full veterinary daily care of the dogs included in this study. Previously harvested dog myocardial tissues for electron microscopy were used for this study.

### 4.2. Light and Electron Microscopy

Paraffin sections 6 μm thick were cut and stained with hematoxylin and eosin (H&E, Sigma-Aldrich, St. Louis, MO, USA) and underwent immunohistochemistry for mouse anti-metallothionein E 9, 1:100 (DAKO Corporation M0639, Carpinteria, CA, USA). Two board-certified pathologists without access to the identification codes reviewed the sections.

### 4.3. Examination of Heart Samples by Transmission Electron Microscopy (TEM)

Heart ultrastructural changes were assessed by three experienced pathologists and two electron microscopists blind to the study group. Electron microscopy was performed in 8 human age-matched cases: 4 northern and 4 southern MCMA and in 5 southern MCMA dogs. Tissues were post-fixed in 1% osmium tetraoxide and embedded in Epon. Semi-thin sections (0.5 to 1 μm) were cut and stained with toluidine blue for light microscopic examination. Ultra-thin sections (60–90 nm) were cut and collected on slot grids previously covered with formvar membrane. Sections were stained with uranyl acetate and lead citrate, and examined with a JEM-1011 (JEOL, Osaka, Japan) microscope. Each electron micrograph was evaluated separately, and then compared by group.

### 4.4. Estimation of mRNA Abundance by RT-PCR

To determine the expression of mRNA from PrP^C^ and BiP, total RNA was extracted from the heart samples using Trizol Reagent (InVitrogen Corp., Carlsbad, CA, USA). RNA integrity, concentration, and purity were determined by spectrophotometry using the NanoDrop ND-1000 (Nanodrop Corp., Wilmington, DE, USA), keeping only samples with the OD A260/A280 and the OD A260/A230 ratios close to 2.0. Small fragments of myocardium, while in ice, were homogenized in 1 mL of Trizol, and the tissue homogenate was centrifuged at 12,000× *g* for 15 min at a temperature of 4 °C. The primers were: PrP^C^ forward: 5′-GTGCACGACTGCGTCAAT-3′, reverse: 5′-CCTTCCTCATCCCACTATCAGG-3′ sequences reported in the GenBank depository. The results were normalized using housekeeping gene GAPDH forward: 5′-ATGATCTTGAGGCTGTTG-3′, reverse: 5′-CTCAGACACCATGGGGAA-3′ [45]. The primers for the human BiP were: forward: 5′-CGAGGAGGAGGACAAGAAGG-3′, reverse: 5′-CACCTTGAACGGCAAGAACT-3′ [38].

The PCR conditions were 1 cycle of 10 min at 95 °C, 40 cycles at 95 °C (30 s), 55 °C (60 s) and 72 °C (60 s), followed by one cycle at 55 °C and 95 °C (30 s). The difference in the cycle threshold (Δ*C*_T_) value was derived by subtracting the *C*_T_ value for GAPDH which served as the internal control, from the *C*_T_ value for BiP and PrP^C^. All reactions were run in duplicates using a Stratagene MX 3005P machine (Agilent Technologies, Inc, Santa Clara, CA, USA). Relative quantitation of gene expression was performed by the 2^−ΔΔ^*^C^*^(T)^ method described by Livak and Schmittgen [78], values for both genes were expressed as a several fold increase (>2) according to the formula 2 Δ*C*_T_ (high pollution) – Δ*C*_T_ (low pollution).

### 4.5. Statistics

Statistical analyses were performed using the SAS statistical software 9.0 version (SAS, Cary, NC, USA). The student’s *t* test, the sign test and/or the Wilcoxon signed rank test were used to test whether there were significant differences in the expression of the selected genes. Pearson’s r statistic was carried out for transformed data, while Kendall’s *tau* was carried out for original data. Both tests were carried out to evaluate the correlation between the target genes ventricular expression, the residency and age effects. Significance was assumed at *p* < 0.05. Data are expressed as mean values ± SD.

### 4.6. Air Pollution Data

Fine particulate matter data from the targeted monitoring stations in Mexico City Metropolitan Area (MCMA) were obtained for the 1997–2012 period. The selection of the control subjects was made on the basis of their residency in cities characterized by clean environments with concentrations of the six criteria air pollutants (ozone, particulate matter, sulfur dioxide, nitrogen oxides, carbon monoxide and lead) below the current US EPA standards [79].

## 5. Conclusions

In summary, exposure to air pollution produces differential ventricular up-regulation of key genes involved in the protection against oxidative stress and metal toxicity and genes up-regulated in response to endoplasmic reticulum stress. The integrity of ER/SR is critical for proper protein quality control machinery, and sustained ER stress in a highly dynamic organ could result in detrimental irreversible consequences [34,39,58,59]. We fully expect significant changes in PrP^C^ and GRP78/BiP as urbanites age and compensatory defense mechanisms decrease, along with the development of clinical CV disease. Long exposure to environmental or man-made nanosized PM likely increases the risk for cardiovascular damage, morbidity and mortality with reductions in life expectancy. Emphasis should be placed upon the potential role of endoplasmic reticulum stress in the initiation and progression of cardiac and vascular dysfunction and the discussion of possible strategies to target this pathway toward the development of cardioprotective interventions. Since the myocardial alterations are observed very early in highly exposed children, the next phase of research ought to include investigation of multi-domain cardioprotective pediatric strategies [11].

## Figures and Tables

**Table 1.** PM_2.5_ annual concentrations in μg/m^3^ for the North *vs.* the South selected monitoring stations. The differences are significant with a *p* < 0.0001.

Year	Pedregal		Xalostoc	
	
	Mean	SD	Mean	SD
1997	21.6	16.6	71.3	34.1
1998	29.3	16.8	64.9	25.4
1999	24.4	9.2	71.0	26.6
2000	24.7	11.3	54.8	25.3
2001	23.6	10.1	41.1	17.2
2002	23.1	9.7	38.0	13.7
2003	23.4	11.3	41.8	14.4
2004	18.4	9.4	35.5	14.7
2005	20.9	11.5	30.4	17.1
2006	17.8	8.4	29.8	15.6
2007	16.2	8.5	25.3	11.3
2008	18.0	8.3	26.3	10.0
2009	18.4	8.7	26.4	10.7
2010	14.4	7.4	24.9	13.2
2011	16.7	8.3	24.7	11.5
2012	17.0	7.5	25.9	11.7

**Table 2.** RT-PCR results in Mexico City subjects expressed as mean ± SD fold increase *vs.* controls for PrP^C^ and BiP/GRP78 in right and left ventricles. PrP^C^ left ventricular expression is significantly different from controls (*p* = 0.006). BiP/GRP78 left and right ventricular expression is significantly different from controls. There is a significant difference in the expression of PrP^C^ between the left and the right ventricles in Mexico City subjects (*p* < 0.0001).

RT-PCR	PrP^C^ left ventricle	PrP^C^ right ventricle	BiP left ventricle	BiP right ventricle
Mean	6.852	0.7209	3.410	2.465
Std. deviation	7.681	0.7207	2.254	1.794
*p* value exposed *vs.* controls	0.0062	0.1360	0.00004	0.0007
*p* value left *vs.* right ventricles in exposed subjects	<0.0001		0.1189	

**Table 3.** Correlation tests using Pearson’s *r* statistic for transformed data and Kendall’s *tau* for original data.

Pearson and Kendall’s Stats	Pearson’s *r* PrP^C^_L & BiP_L	Pearson’s *r* PrP^C^_R & BiP_R	Kendall’s *tau* PrP^C^_L & BiP_L	Kandall’s *tau* PrP^C^_R & BiP_R
*r*	0.6645	0.2409	0.6230	0.1530
*p* value	5.435 × 10^−4^	0.3063	3.334 × 10^−5^	0.3465

**Table 4.** Association test *p* values results for region of residency: PrP^C^ and BiP/GRP78 expression in left and right ventricles for North *vs.* South Mexico City residents.

**Two-sample test**				

	PrP**^C^**_L	PrP**^C^**_R	BiP_L	BiP_R
*p* value	0.1272	0.9024	0.4563	0.6817

**Wilcoxon rank sum test**				

	PrP**^C^**_L	PrP**^C^**_R	BiP_L	BiP_R
*p* value	0.3050	0.8366	0.2973	0.9864

**Table 5.** Children PrP^C^ and GRP78/BiP fold increase results for left ventricle (LV) and right ventricle (RV), North *vs.* South residency.

	PrP^C^ LV North	PrP^C^ LV South	PrP^C^ RV North	PrP^C^ RV South	BiP LV North	BiP LV South	BiP RV North	BiP RV South
Mean	8.548	9.292	0.9723	0.5889	4.905	3.317	3.151	1.542
Std. deviation	9.151	11.04	1.149	0.6262	2.720	1.355	2.333	0.8514
*p* value	0.5273		0.9273		0.4970		0.3825	
